# Prevalence of depressive symptoms and associated factors among patients with tuberculosis attending public health institutions in Gede’o zone, South Ethiopia

**DOI:** 10.1186/s12889-020-09794-z

**Published:** 2020-11-13

**Authors:** Kalkidan Yohannes, Hirbaye Mokona, Lulu Abebe, Mohammed Feyisso, Adane Tesfaye, Getachew Tesfaw, Getinet Ayano

**Affiliations:** 1 Department of Psychiatry, College of Medicine and Health Sciences, Dilla University, P.O. Box 419, Dilla, Ethiopia; 2 School of Public Health, College of Medicine and Health Sciences, Dilla University, Dilla, Ethiopia; 3 Department of Psychiatry, College of Medicine and Health Sciences, University of Gondar, Gondar, Ethiopia; 4 Research and Training Department, Amanuel Mental Specialized Hospital, Addis Ababa, Ethiopia; 5 School of Public Health, Curtin University, Bentley, Perth, Western Australia

**Keywords:** Depressive symptoms, Prevalence, Tuberculosis, Ethiopia

## Abstract

**Background:**

Depression is a common mental disorder among patients with tuberculosis and it is associated with a greater risk of suicide, multidrug-resistant tuberculosis, and poor quality of life. Evidence suggests the early identification of depression among patients with tuberculosis is important to decrease adverse outcomes. However, there are limited studies that examined the prevalence and determinants of depressive symptoms among patients with tuberculosis. This study aimed to assess the prevalence and associated factors of depressive symptoms among patients with tuberculosis attending public health institutions in Gede’o zone, South Ethiopia.

**Methods:**

An institution-based a cross-sectional study was conducted from November 1 to December 30, 2018, among a randomly selected sample of 415 patients with tuberculosis attending public health institutions in Gede’o zone, South Ethiopia. Patient Health Questionnaire (PHQ-9) was used to assess depressive symptoms. Logistic regression was used to identify the potential risk factors of depressive symptoms. The strength of the association was presented by crude odds and adjusted odds ratio with their corresponding 95% CI. Finally, the statistical significance was set at *p* < 0.05.

**Results:**

The prevalence of depressive symptoms was found to be 45.5% (95% CI; 41.1–50.1%) among patients with tuberculosis; 33.3% had moderate, 9.8% had moderately severe, and 2.4% had severe depression. After adjusting for the possible confounders, being on re-treatment for tuberculosis (AOR = 2.47, 95% CI: 1.17–5.22), aged ≥45 years (AOR = 2.41, 95% CI: 1.09–5.32), having poor social support (AOR = 4.21, 95% CI: 2.10–8.47), and Tuberculosis/Human Immunodeficiency Virus (TB/HIV) co-infection) (AOR = 3.96, 95% CI 2.0, 7.84) were significantly associated with depressive symptoms among patients with TB.

**Conclusions:**

This study suggests that a substantial percentage of patients with TB had depressive symptoms (45.5%). TB/HIV coinfection, being on re-treatment for tuberculosis, those having poor social support, patients aged 45 and above were factors associated with depressive symptoms. Routine screening of depression among patients with TB is warranted. Moreover, patients with TB falling into the risk categories should be more carefully monitored for depression and when possible referred to mental health professionals.

## Background

Depression is a common and serious mental disorder, characterized by persistent sadness and a loss of interest in activities that you normally enjoy [1]. Aside from the emotional problems caused by depression, individuals can also present with a physical symptom [1–3]. It is also accompanied by an inability to carry out daily activities, for at least two weeks [1, 3]. This disorder is a serious, recurrent one, which erodes the quality of life and fulfillment of social and familial roles [4], and the World Health Organization (WHO) ranks depression as the fourth leading cause of disability worldwide (8). It is the leading mental health-related cause of the Global Burden of Disease (GBD) [5]. According to the findings from the National Health Survey data, the prevalence of depressive episode in Ethiopia was 9.1% [6]. Depression is a common comorbid condition of patients with TB [7] and it is associated with higher poor clinical outcomes [8].

TB is a widely spread infectious disease caused by the bacterium Mycobacterium tuberculosis. It typically affects the lungs (pulmonary TB), but can also affect other sites (extra-pulmonary TB) [9]. TB continues to remain a major health problem, especially in the developing world [10]. Despite the availability of effective chemotherapy, TB killed 1.3 million people in 2017. Globally, the best estimate is that 10 million people (range, 9-11.2 million) equivalent to 133 cases (range, 120–148) per 100,000 population developed TB disease in 2017: 5.8 million men, 3.2 million women and 1.0 million children [11]. According to the 2014 global report on TB published by the World Health Organization (WHO), Ethiopia has achieved all the three targets of the Millennium Development Goals (MDG) regarding TB prevention and controls [12].According to the report, the incidence rate of TB is falling significantly and the prevalence and the death rate due to TB have declined by more than 50% [12].

There is a dearth of data on the prevalence of depressive symptoms in TB patients in Ethiopia, however, a study conducted in Wolaita Sodo reported a 43.4% prevalence of depression in TB patients [13]. A study conducted in the Jimma University Specialized Hospital and Jimma Health Center revealed 49% of depression prevalence among TB patients [14]. Furthermore, another study in Butajira showed a high prevalence of probable depression, 54.0% among newly diagnosed TB patients [15].

Co-occurrence of TB and depression is associated with a range of adverse health outcomes, including functional impairment, increase medical costs, the emergence of multidrug-resistant TB and poor health-related quality of life [7, 16–19].Some evidence suggests that among TB patients, there were high prevalence rates of psychiatric comorbidity, especially depression, as well as specific psychological reactions and reviews indicating psychiatric complications as adverse effects of anti-TB medication [7, 20].

Multiple factors can affect depression among TB patients, including socio-demographic and economic factors: age of the patient, being female, marital status, income, occupation, perceived social support, perceived stigma to physical conditions, TB-HIV co-infection, hazardous alcohol use, and physical symptoms [13, 21–23].

Measurement of depression in TB patients is essential to have an in-depth understanding of the effect of disease on the mental health of the patient. In Ethiopia, there is limited literature regarding depression and important contributing factors like hazardous alcohol use, among TB patients. Therefore, this study aimed to assess the prevalence of depressive symptoms and associated factors among TB patients attending TB clinics of public healthcare institutions in Gede’o zone, South Ethiopia. The findings from this study will assist the national TB program to develop effective intervention strategies for TB patients with problems related to depression. Hence, the finding of this study will address a gap in formulating the necessary solutions.

## Methods

### Study design and period

An institution-based cross-sectional study was conducted from November 1 to December 30, 2018.

### Study site

The study was conducted among adult patients attending a TB clinic in public health institutions of Yirgacheffe, Wonago and Dilla Zuriya districts, Gedeo zone, South Ethiopia. Gedeo zone is found in South Nations, Nationalities and Peoples’ Regional States of Ethiopia, 359 km southeast of Addis Ababa (the capital city of Ethiopia). In the zone, there is one referral hospital, two primary hospitals, and 39 health centers. Except Dilla University Referral Hospital, none of them had mental health professional or no expert was participating in the Mental Health Gap Action Program (mh-GAP) training for primary care providers.

### Sample size determination and sampling procedure

The sample size calculated using assumptions: margin of error 5, 95% CI, the estimated prevalence of depression, 43.4% from a study in Wolaita Sodo, Ethiopia [13] and the non-response rate of 10%. The final calculated sample size was 415. Study participants were proportionally allocated for each health facility, according to patient flow by referring to the previous year’s annual reports. A systematic sampling technique was used to select study participants. Sampling interval was determined by dividing the total study population who had follow-up during the four-week data collection period by the total sample size, then the starting point was randomly selected.

### Study population

All adult TB patients who were attending TB clinics of public health institutions in the Gede’o zone were studied populations. Patients aged 18 to 65 years old and patients who were on anti-TB medication for at least 2 weeks were included in the study. Patients who were severely ill during the study period were excluded.

### Data collection

Data was collected by six clinical nurses who were assisted by two public health officers, working in the TB clinic of the respective study health institutions.

### Data collection instruments

Interviewer-administered questionnaire was used to collect data, which contains data on socio-demographic characteristics of the participants, psychosocial characteristics, clinical characteristics, alcohol related characteristics, and data on the outcome variable (depressive symptoms).

#### Perceived social support

Data on social support was assessed by Oslo social support scale [24]. It is a three items social support scale (OSS-3. It covers different fields of social support by measuring the number of people the respondent feels close to, the interest and concern showed by others and the ease of obtaining practical help from others. It has the sum score scale ranging from 3 to 14 with three broad categories: “poor support” 3–8, “moderate support” 9–11 and “strong support” 12–14 [24].

#### Perceived TB stigma scale

Data on TB stigma felt by the patients was assessed by the TB stigma scale. It is 11–item scale that is used to assess the stigma felt by TB patients [25]. The instrument was adopted and translated to Amharic language and highly reliable in a pre-test (Cronbach’s α = 0.89). Item scores of the stigma questions were summed to construct a single stigma variable. Participants were classified as having or not having perceived stigma using the mean of the stigma variable as a cut-off point [25, 26].

#### Hazardous alcohol use

Data on hazardous alcohol use was collected through the Alcohol Use Disorders Identification Test (AUDIT) [27]. It is a brief screening instrument designed for the early detection of hazardous and harmful alcohol use in a variety of settings. It focuses on both the past and current alcohol drinking. Each item is rated using item-specific anchors scored 0 to 4 and summed for a total score of 0 to 40. A score of 8 or more is considered to indicate hazardous alcohol use. The AUDIT has been validated across genders and in a wide range of racial/ethnic groups and is well-suited for use in primary care settings [27].

#### Depressive symptoms

Data on the outcome variable was collected through interviews using a standard questionnaire, Patient Health Questionnaire (PHQ-9) which is a 9-item depression screening and diagnostic questionnaire for major depressive disorder based on the Diagnostic Statistical Manual-IV criteria with sensitivity 86% and specificity 67% [28]. The PHQ-9 appears to be a reliable and valid instrument and has been validated and used in Ethiopia. A score of 10 or more out of 27 indicates the presence of depressive symptoms. For severity classification, a score of 10–14 indicates ‘moderate depression’, 15–19 indicates ‘moderately severe depression’ and 20–27 indicates ‘severe depression’ [28].

### Data processing and analysis

Data were cleaned, coded and entered to EPINFO version 7 and analyzed using SPSS-20 respectively. Using descriptive methods, the data were summarized and the estimated prevalence of depressive symptoms was determined. Associations of depressive symptoms and its factors were identified using logistic regression analyses. Following each bivariate regression, multivariable logistic regression models were constructed. The statistical significance was set at *p* < 0. 05.

## Results

### Socio-demographic and economic characteristics of the participants

A total of 409 participants took part in the present study (response rate 98.6%) and the majority of the participants (61.6%) were men. The mean age of the participant was 31.9 years (SD = 11.85) and 53.1% were married. More than one-third (40.3%) of the participants had attended primary school, (53.1%) lives in rural areas, and the vast majority of the participants (78.2%) earn less than 1539 ETB (54.19 USD) per month (Table 1).

**Table 1:** Distribution of tuberculosis patients attending tuberculosis unit of public health institutions, Gede’o zone, South Ethiopia, 2018 (*n* = 409)

Variables	Categories	Frequency	Percent (%)
Sex	Male	252	61.6
	Female	157	38.4
Age in years	18–29	216	52.8
	30–39	98	24.0
	40–49	56	13.7
	50 and above	39	9.5
Marital status	Married	217	53.1
	Single	157	38.4
	Divorced	23	5.6
	Widow	12	2.9
Level of education	No formal education	92	22.5
	Primary education	165	40.3
	Secondary education	98	24.0
	College and above	54	13.2
Occupational status	Employed	69	16.9
	Farmer	114	27.9
	Merchant	89	21.8
	Unemployed	137	33.5
Place of residence	Rural	217	53.1
	Urban	192	46.9
Average monthly income	< 1539 ETB (< 56.13 USD)	320	78.2
	> = 1539 ETB(> = 56.13 USD)	89	21.8

### Clinical characteristics of the participants (patients with TB)

Of the 409 participants, 302 (73.8%) were pulmonary TB patients, 250 (61.6%) were in the intensive phase of the treatment, and 15.2% were re-treatment cases. One in five participants (20%) were hazardous alcohol users, 71 (17.4%) had comorbid HIV, and 22 (5.4%) had co-morbid chronic conditions. In two hundred thirty-six (57.7%) of the TB patients, the illness had a duration of 6–12 months (Table 2).

**Table 2:** Description of clinical and alcohol-related characteristics of tuberculosis patients attending the tuberculosis unit of public health institutions, Gede’o zone, South Ethiopia, 2018 (*n* = 409)

Variables	Categories	Number (%)
Classification of TB	Pulmonary	302 (73.8)
	Extra-pulmonary	107 (26.2)
Phase of treatment	Intensive phase	250 (61.1)
	Continuation phase	159 (38.9)
Treatment status	New case	347 (84.8)
	Re-treatment case	62 (15.2)
Comorbidity	TB/HIV comorbidity	71 (17.4)
	Other comorbid condition	22 (5.4)
	No comorbidity	316 (77.2)
Family history of mental Illness	Yes	23 (5.6)
	No	386 (94.4)
Duration of illness	< 6 months	78 (19.1)
	6–12 months	236 (57.7)
	> = 12 months	95 (23.2)
Hazardous alcohol use	No	327 (80)
	Yes	82 (20)

### Psychosocial characteristics of the respondents

**Fig. 1:**
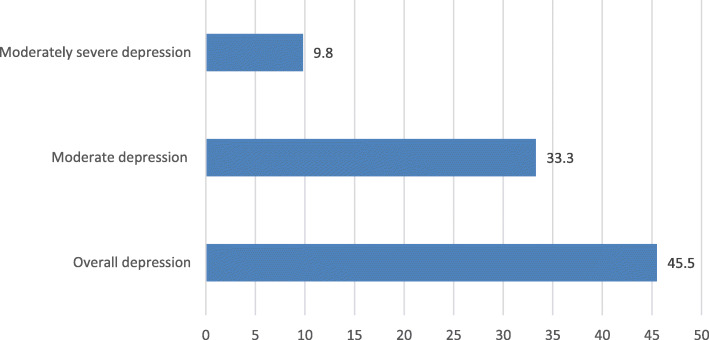
Percentage distribution of perceived social support of tuberculosis patients attending tuberculosis unit of public health institutions, Gede’o zone, South Ethiopia, 2018 (*n* = 409)

Among the total 409 participants, 139 (34%) had poor social support and 177 (43.3%) had intermediate social support. Regarding perceived TB stigma, more than one-third of the participants (38.9%) had perceived TB stigma (Fig. 1).

### Prevalence of depressive symptoms

**Fig. 2:**
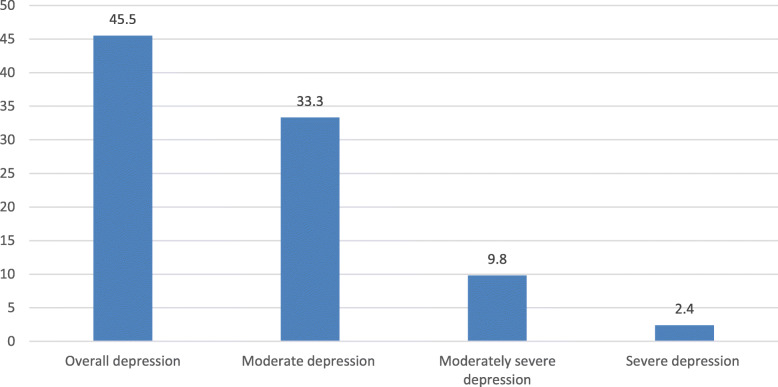
Percentage distribution of depression severity among tuberculosis patients attending tuberculosis unit of public health institutions, Gede’o zone, South Ethiopia, 2018 (*n* = 409)

The prevalence of depressive symptoms was 45.5% (95% CI = 41.1–50.1); 33.3% had moderate, 9.8% had moderately severe, and 2.4% had severe depressive symptoms (Fig. 2).

### Factors associated with depressive symptoms among TB patients

#### Bivariate analysis

In this study, age, marital status, having perceived TB stigma, poor social support, phase of treatment, hazardous alcohol use, medical comorbidity, duration of illness and treatment status were significantly associated with depressive symptoms among TB patients in bivariate analysis (Table 3).

**Table 3:** Bivariate and multivariable analysis of factors associated with depressive symptoms among tuberculosis patients attending tuberculosis unit of public health institutions, Gede’o zone, South Ethiopia, 2018 (*n* = 409)

VARIABLES	CATEGORIES	DEPRESSION		COR (95%CI)	AOR (95% CI)
		Yes	No		
Marital status	Married	104	113	1	1
	Single	56	101	0.60 (0.39, 0.91)	0.81 (0.47, 1.41)
	Divorced/ separated	26	9	3.13 (1.40, 7.01)	0.85 (0.32, 2.23)
Age of participants	18–24 years	39	96	1	1
	25–34 years	62	60	2.54 (1.52,4.25)	1.65 (0.86, 3.17)
	35–44 years	48	37	3.19 (1.81, 5.64)	1.93 (0.89, 4.16)
	45 and above years	37	30	3.03 (1.65, 5.58)	2.41 (1.09, 5.32) *
Hazardous alcohol use	Yes	55	27	3.04 (1.82,5.08)	1.65 (0.91, 3.01)
	No	131	196	1	1
Comorbidity	TB/HIV coinfection	53	18	4.94 (2.76, 8.83)	3.96 (2.0, 7.84) ***
	Another comorbidity	15	7	3.59 (1.42,9.07)	2.92 (0.97, 8.77)
	No comorbidity	118	198	1	1
Duration of illness	< 6 months	23	55	1	1
	6–12 months	111	125	2.12 (1.22, 3.68)	1.72 (0.90, 3.31)
	> = 12 months	52	43	2.89 (1.53, 5.58)	0.92 (0.40, 2.14)
Perceived social support	Poor support	99	40	7.53 (4.14, 13.68)	4.21 (2.10, 8.47) ***
	Intermediate support	64	113	1.72 (0.98, 3.02)	1.22 (0.63, 2.36)
	Strong support	23	70	1	1
Treatment status	New case	139	208	1	1
	Re-treatment case	47	15	4.68 (2.52, 8.71)	2.47 (1.17, 5.22) *
Perceived TB stigma	Yes	78	81	1.26 (0.84, 1.88)	1.34 (0.81, 2.21)
	No	108	142	1	1
Phase of treatment	Intensive	98	152	1	1
	Continuation	88	71	1.92 (1.28, 2.87)	1.69 (0.98, 2.90)

Note; A *p-value less than 0.05; **p-value less than 0.01; ***p-value less than 0.001 1 = reference *P* value of Hosmer and Lemeshow Test = 0.56

#### Multivariable analysis

In the final and fully adjusted multivariable model, TB/HIV co-infection, being on re-treatment for TB, poor social support, patients aged 45 and above were found to be significantly associated with depressive symptoms among TB patients.

The odds of having depressive symptoms for those patients with TB/HIV coinfection was 3.96 fold higher than those patients who had no TB/HIV coinfection (AOR = 3.96, 95% CI 2.0, 7.84). Regarding social support, those who had poor social support were more than 4 times more likely to develop depressive symptoms than those who have intermediate and strong social support (AOR = 4.21, 95% CI: 2.10–8.47).

The study also found that the odds of developing depressive symptoms among those who were on re-treatment for TB were 2.47 times higher as compared to those who were on the new TB treatment category (AOR = 2.47, 95% CI: 1.17–5.22). Those patients aged 45 and above were at 2.41 times higher odds of developing depressive symtoms when compared with those aged 18–24 years (AOR = 2.41, 95% CI: 1.09–5.32) (Table 3).

## Discussion

In this study, we found that 45.5% of patients with TB had depressive symptoms, which is consistent with the reported prevalence from previously published studies from Ethiopia (43.4% in Wolaita zone [13], and 49% Silte and Gurage zone among New TB cases [15]), Nigeria (45.5%), Pakistan (49.4%) and China (48.0%) [13, 14, 29–32].

However, the study prevalence estimates are lower than those reported by prior studies from Cameroon, South Africa, Pakistan, Kashmir, and India, which ranges from 51.9 to 84% [15, 33–40]. The differences in the prevalence of depression across the studies could be attributable to several factors, including differences in the sample size, the population being studied, screening tools used, different cut-off scores employed, the study period, classification of tuberculosis and treatment status of TB patients who were in follow up.

Contrarily, the finding of this study was higher compared to the results from studies conducted in Nigeria (27.7%) [41], Philippines (16.8%) [22], and India (23.6%) [42]. The possible explanation for the observed differences could be the difference in population characteristics, the time of assessment (including severity, phases of Tb treatment and being on TB medicine or not), as well as the difference in the instruments used to measure depression.

Concerning associated factors, in this study, social support was significantly associated with depressive symptoms among TB patients. Those who had poor social support were more than 4 times more likely to develop depressive symptoms than those who have intermediate and strong social support. The finding was supported by previous epidemiological studies conducted in Ethiopia and India [13, 32]. The possible explanation to account for this finding lies in the notion that psychological support is a highly important protective factor against depression in adults with chronic illness. Social support, such as having someone to confide in, attempts to directly reduce the negative feelings associated with a distressing situation [43]. Thus, perceived poor social support diminishes feelings of closeness and being cared for by the family members, and hence compromises individuals’ ability to seek help and advice from families and neighbors. On the other hand, having good social support allows the individual to look at the positive aspects of life, thereby resisting the development of negative emotions such as depression [44].

Regarding the comorbid illness, the odds of having depression for those patients with TB/HIV coinfection was 3.96-fold higher than those patients who had no TB/HIV coinfection. This finding was consistent with previous studies [13, 14, 34]. The dual burden of TB/HIV coinfection among TB patients escalates the probability of poor mental health. In addition to this, the depressive symptoms might be due to the combined side effects of some anti-TB and antiretroviral drugs on the mental health of the patients. Some anti-HIV drugs can affect mental health. Most notably, the non-nucleoside reverse transcriptase inhibitor (NNRTI) efavirenz (Sustiva, also in the combination pill, Atripla) has been associated with depressive symptoms [41, 45].

Treatment status was the other factor that was found to be significantly associated with depressive symptoms among TB patients. The odds of developing depressive symptoms among those who were on re-treatment for TB were 2.47 times higher as compared to those who were on the new TB treatment category. This is consistent with the results reported from studies conducted in Cameroon [34]. The possible explanation to account for this finding lies in the notion that having a previous history of treatment failure, default and relapse to be more prone to worsening of physical condition as well as poor mental health or the depression may predispose patients to do not adhere to their TB medication, which may lead to failure, relapse or default [34].

Finally, those patients aged 45 and above were at 2.41 times higher odds of developing depressive symptoms when compared with those aged 18–24 years. This finding is consistent with findings from previous studies [14, 21, 29]. Although it is difficult to determine causality, it is common to find people who were aged 40 and above to be more prone to depressive symptoms, possibly due to poor social support, family responsibility, loss of a spouse or compromised immunity due to the comorbid illness [21, 29].

### The strength and limitation of the study

The study has several strengths. First, the study included important variables that were not included in previous studies. Second, we used the standardized instrument for measuring depression (patient Health Questionnaire-9 (PHQ-9). Third, we used a validated and standardized tool for the assessment of independent variables such as alcohol use by Alcohol Use Disorder Identification Test (AUDIT), and TB stigma scale.

A limitation of the study is that the probability of overestimation of the prevalence of depressive symptoms due to the fact that the PHQ-9 has some biological symptoms of TB. Another limitation is due to the design. Since the cross-sectional study design could not show clear risks of depression in TB patients, it was difficult to imply the temporal relationship between significantly associated factors and depression. Additionally, due to the nature of the study design, the researchers cannot distinguish whether the reported prevalence is due to pre-existing or new-onset cases (depressive symptoms).

## Conclusion

In this study, a considerable percentage of patients with TB had depressive symptoms (45.5%). Having TB/HIV co-infection, being on re-treatment for TB, those having poor social support, and patients aged 45 and above were found to be significantly associated with depressive symptoms. Therefore, routine screening of depressive symptoms among patients with TB is recommended. Attention needs to be paid for patients having TB/HIV co-infection, being on re-treatment for TB, those having poor social support, and patients aged 45 and above. Moreover, TB patients falling into the risk categories should be more carefully monitored for depressive symptoms and when possible referred to mental health professionals.

## Data Availability

The datasets used and analyzed during the current study are available from the corresponding author on reasonable request.

## Abbreviations

AUDIT: Alcohol use disorders identification test
PHQ-9: Nine-item patient health questionnaire
MDR-TB: Multiple drug resistance-tuberculosis
NNRTI: Non-nucleoside reverse transcriptase inhibitor
SD: Standard deviation
TB: Tuberculosis
YLD: Years lived with disability
